# Galvanizing Collective Action to Accelerate Reductions in Maternal and Newborn Mortality and Prevention of Stillbirths

**DOI:** 10.9745/GHSP-D-20-00575

**Published:** 2021-06-30

**Authors:** Anita Gibson, Lisa Noguchi, Mary V. Kinney, Hannah Blencowe, Lynn Freedman, Tlaleng Mofokeng, Mickey Chopra, Queen Dube, David Ntirushwa, Angela Nguku, Anshu Banerjee, Swaraj Rajbhandari, Hadiza Galadanci, Martina Lukong Baye, Pashtoon Zyaee, Lia Tadesse, Dalya Eltayeb, Aparajita Gogoi, Shams El Arifeen, Samba Sow, Patrick Kuma-Aboagye

**Affiliations:** a AlignMNH Secretariat, Jhpiego, Baltimore, MD, USA.; b School of Public Health, University of the Western Cape, South Africa.; c Centre for Maternal, Adolescent, Reproductive & Child Health, London School of Hygiene & Tropical Medicine, London, United Kingdom.; d Columbia University Mailman School of Public Health, New York, NY, USA.; e United Nations Human Rights Council on the Right to Health, Commission for Gender Equality, South Africa.; f Health, Nutrition and Population Global Practice, World Bank, Washington, DC, USA.; g College of Medicine, University of Malawi, Zomba, Malawi.; h Department of Obstetrics and Gynecology, Centre Hospitalier Universitaire de Kigali, Kigali, Rwanda.; i White Ribbon Alliance, Kenya.; j World Health Organization, Geneva, Switzerland.; k Nidan Hospital, Lalitpur, Nepal.; l African Center of Excellence in Population Health and Policy, Nigeria.; m Ministry of Public Health, Yaounde, Cameroon.; n Advisory Board, Afghan Midwives Association, Kabul, Afghanistan.; o Ministry of Health, Addis Ababa, Ethiopia.; p Federal Ministry of Health, Khartoum, Sudan.; q Center for Catalyzing Change, Delhi, India.; r icddr,b, Dhaka, Bangladesh.; s Center for Vaccine Development, Bamako, Mali.; t Ministry of Health, Accra, Ghana.

## Abstract

With less than 10 years remaining to achieve the sustainable development goals, there is an urgent need for collective action to accelerate progress for maternal and newborn health and prevention of stillbirths. We outline a new global initiative, AlignMNH, designed to create opportunities to better align efforts and drive improvements.

## Key Messages

- Historically, global efforts to accelerate progress toward achieving health and well-being for women and children have appeared separate, despite the inextricable links among maternal, perinatal, and newborn health outcomes.
- Accelerating progress demands that the maternal and newborn health (MNH) community more rapidly and effectively share learning, new evidence, and program experiences.
- A new global initiative, AlignMNH, will establish a country-driven, multidirectional knowledge hub and series of convenings to promote purposeful knowledge sharing and problem solving and align on priorities for action. These efforts are intended to facilitate increased application of evidence and regular review of progress ultimately contributing to improved MNH and prevention of stillbirths.

## ENSURING MATERNAL AND NEWBORN HEALTH REMAINS A PRIORITY

Every day, there are an estimated 810 maternal and 7,000 newborn deaths, and more than 5,000 stillbirths, most of which are preventable.^1^^-^^3^ While progress has been made in reducing maternal and neonatal morbidity and mortality and preventing stillbirths worldwide, inequities and gaps in quality of care persist^4^ and are disproportionately most dire in countries affected by conflict.^5^ In 2020, the coronavirus disease (COVID-19) pandemic and response exposed multiple system vulnerabilities, exacerbated inequities to accessing care, and caused widespread disruption in reproductive, maternal, newborn, and child health services.^6^^,^^7^ Emerging evidence and modeling estimates of the indirect effects of the COVID-19 pandemic on maternal and newborn mortality in low- and middle-income countries (LMICs) reflect a sobering picture of what could lay ahead, with additional deaths estimated to be in the tens of thousands for mothers and hundreds of thousands for stillbirths and children aged under 5 years.^3^^,^ ^8^^-^^10^ A dedicated, focused effort must be made to ensure maternal and newborn health (MNH) and prevention of stillbirths remain a priority.

The world is at a critical inflection point, with less than 10 years remaining to achieve the Sustainable Development Goals (SDGs). MNH risks getting lost amidst multiple competing health priorities as countries transition through different stages of the pandemic response. Several additional risks exist in the near term: human resource constraints, inclusive of midwives;^11^ increased migration of skilled health workers from rural to urban areas and from low- and middle-income to high-income countries; lack of fiscal space leading to increased financial barriers, such as user fees; and the immiseration of populations leading to increased gender oppression and disadvantage. These are just a few examples of risks that must be mitigated to ensure sustained improvements in MNH.

Future progress also requires acknowledging and addressing unequal power structures in the field of global health,^12^ in large part a legacy of colonialism, which have been identified as substantial barriers to the success of country-level MNH implementation agendas for the fulfillment of human rights and the provision of comprehensive, high-quality care.^13^^,^^14^ The global MNH community must align on priorities to address inequities fueled by lack of just representation of citizens' voices and country needs and professional hierarchies. Resources must be directed to recover previous gains and to accelerate progress toward equitable, accessible, high-quality care for all women and newborns. Addressing these multiple challenges and sustaining progress will not be achieved within the MNH community alone and will require alliances with other communities both globally and nationally.^15^^,^^16^

Historically, global efforts to accelerate progress toward achieving health and well-being for all women and children have appeared separate, despite the inextricable links among maternal, perinatal, and newborn health outcomes. For maternal health, the Safe Motherhood Call to Action in 1987 signaled the need to focus on reducing maternal mortality, highlighting the disproportional burden of mortality in LMICs.^17^ In 2000, the establishment of a Millennium Development Goal (MDG) specific to maternal mortality kept a spotlight on the need to invest in the health of women. The MDGs did not explicitly include newborn mortality and stillbirths. To put a focus on these deaths, the World Health Assembly endorsed the Every Newborn Action Plan (ENAP) in 2014. ENAP set goals for ending preventable newborn mortality and stillbirths with coverage targets, strategic objectives, and milestones to 2020.^18^ In 2015, the World Health Organization (WHO) released *Strategies Toward Ending Preventable Maternal Mortality* (EPMM), which outlined global targets and strategies for reducing maternal mortality under the SDGs.^19^ The EPMM working group subsequently released a comprehensive monitoring framework to track progress toward achievement of EPMM targets and priorities.^20^ This work has outlined a way forward amidst the backdrop of changing trends in population demographics and global disease burden.

## ADDRESSING THE NEED FOR A COLLABORATIVE, INTEGRATED APPROACH

To address the perceived and actual divides between MNH communities,^21^ a group of over 50 experts convened in September 2014 to identify specific strategies to improve quality of care and increase collaboration.^22^ Recommended actions focused on data and measurement, commodities, advocacy, human resources, standards for care, technical support, and funding to better integrate maternal and newborn care at multiple levels: service delivery, national policy and programs, and among donors and partners. In 2015, as the world was transitioning from the MDGs with separate goals for mothers and children to the SDGs with a focus on universal health coverage, equity, and integration, more than 1,000 delegates from over 75 countries came together in Mexico City to reflect on emerging evidence and learning from country experiences to forge a way forward to improve MNH.^23^ Ten critical actions were outlined as part of a roadmap in the post-2015 era (Box 1). Five years on, collective action to build on these discussions has been inadequate to drive sufficient progress.

Box 1 **Roadmap for Maternal and Newborn Health in the Post-2015 Era: 10 Critical Actions (Mexico City)^23^**

1. Countries where political leadership acts on strong scientific evidence and the public demands better maternal newborn survival make progress. Governments and societies of countries lagging behind are morally obliged to embrace and implement an active and evidence-based maternal newborn health (MNH) agenda and continuously monitor its progress.
2. Global and national health communities must integrate strategies, services, and funding streams to avoid unnecessary and harmful silos. MNH offers a proven platform to strengthen the entire health system.
3. Weak national health care systems fail too many individuals; reaching the most vulnerable, including adolescents, is an urgent priority.
4. Efforts to improve maternal newborn survival should include attention to maternal morbidities, stillbirths, and child development outcomes; they are essential proxies for inequality and poor quality care.
5. Increasing the investment in better quality MNH services is a fundamental response to health and rights imperatives.
6. Care with dignity does not cost any more. At any resource level, a provider has the opportunity and the obligation to treat clients with compassion and respect.
7. Universal access to integrated sexual and reproductive health care, including contraception, is essential to ensure MNH.
8. It is time to address the gap in measurement, information, and accountability. In order to assess progress in the next 15 years, countries and the global community need to address these complex challenges now.
9. Sharing good news in human development is not a risk but an opportunity to build stronger health programs. This is the time to acknowledge important gains made as strategies are created to implement the SDGs.
10. Supporting all providers, including midwives, to address MNH is imperative to realize the ambitious post-2015 agenda.

Notable efforts have been made toward integrated approaches, namely, the ENAP and EPMM joint metrics agenda, a joint call to action in 2015,^24^ and the release of WHO's *Standards for Improving Quality of Maternal and Newborn Care in Health Facilities.*^25^ Despite these efforts, there is still much work to do to support MNH integration within the health system particularly at the community and primary facility levels, where services are often provided by a single nurse or midwife. Also, the path toward universal health coverage requires bringing primary health care closer to people and including people as active decision makers in their own health. These requirements demand that the health system contribute to creating an environment that is safe and supportive for self-care.^26^

There is still much work to do to support MNH integration within the health system particularly at the community and primary facility levels, where services are often provided by a single nurse or midwife.

Unprecedented opportunities to drive and chart progress now exist. Updated ENAP global, national, and subnational coverage targets and milestones to 2025 were released in September 2020^27^ and EPMM is finalizing a complementary process to establish coverage and milestones for maternal health.^28^ The burden of stillbirths is also now receiving increased attention having previously been largely absent on the global stage.^29^^,^^30^ Stillbirth estimates released in October 2020 provide an important reference to maintain visible and urgent attention on the need for accelerated progress.^3^ We must be vigilant, however, as improvements in effective coverage of interventions do not necessarily reflect who is being left behind.^4^^,^^31^

Financing must support accelerated progress. Considering the multiple categories of SDG financing, both public and private domestic and international funds, multiple paths must be explored to address the finance gaps.^32^ Uncoordinated approaches to funding and limited ability to track resources are potential barriers to more rapid acceleration of achievements in MNH. In recent years, some evidence has suggested that fragmentation, volatility, and transaction costs associated with donor funding have limited the impact of development assistance on health outcomes and sustainability of progress in LMICs,^33^ while other research has not shown any clear relationship between donor proliferation or fragmentation in health sector aid and measures of health service delivery or health outcomes.^34^ The Global Financing Facility (GFF), launched in 2015, was created in an effort to ensure prioritization and financing of health and nutrition for women, children, and adolescents, and provides an opportunity to align domestic and international investments for reproductive, maternal, newborn, child, and adolescent health in several high-burden countries. Increasing linkages between the GFF and country-led processes to monitor progress toward the SDGs may be one path toward better coordination and increased impact of financing and programs, particularly in the setting of narrowing fiscal space for MNH and the damage brought on by the pandemic.

Accelerating progress demands that the MNH community more rapidly and effectively share learning, new evidence, and program experiences. Progress must be tracked, opportunities and gaps must be identified and addressed, and networks to drive and sustain gains must be nurtured. No country is alone in its challenges. Stakeholders in countries must have access to information in ways that are relevant, timely, and actionable. As highlighted in this journal,^21^ focusing on “context-driven, content-focused,” evidence-based solutions is critical to understanding under what conditions something works—and if an approach could be both effective and implementable at scale in another setting. The human rights principles of participation, equality, and nondiscrimination require that priorities are informed by the real, lived experiences of women, and that health systems structures, research methods, and donor funding support this imperative. Within the MNH field, the formal recognition of experience of care as an essential element of quality of care; the burgeoning research, advocacy, and action on respectful care; and the efforts to introduce social accountability mechanisms into health systems are all steps in this direction.^35^^,^^36^ But true transformation to people-centered systems—for both service delivery and policy making—will require concerted, multivalent action and continuing vigilance.

Accelerating progress demands that the MNH community more rapidly and effectively share learning, new evidence, and program experiences.

## FOSTERING MORE COHESIVE EFFORTS TO IMPROVE MNH

To address the need for more cohesion around MNH and prevention of stillbirths, we are committing to a new global initiative, AlignMNH (Box 2). Building on existing assets in the MNH community, AlignMNH will support a predictable cadence of bi-annual international MNH conferences, creating space for stakeholders to review successes, debate the limitations of current strategies, and identify how to address priority issues, questions, and bottlenecks. Complementing the conference series will be a dynamic multidirectional knowledge hub to facilitate a more continuous, virtual dissemination and exchange of evidence and learning to contribute to driving informed action. We openly acknowledge the limitations of online platforms in terms of equitable access to information and advocacy and are committed to better understanding and learning ways to make inclusion in dialogue more equitable and content more reflective of a diversity of experience across countries and stakeholders. AlignMNH will also engage countries to ensure the knowledge hub and conference series are shaped by country priorities and meet their needs to plan, course correct, and drive action powered by data.

Box 2 **AlignMNH Initiative**

Goal: Contribute to accelerated reductions of maternal and newborn mortality and prevention of stillbirths to achieve Sustainable Development Goal era targets and contribute to improved health and well-being in low- and middle-income countries.

Hypothesis: Establishing a country-driven, multidirectional platform to convene the maternal and newborn health (MNH) community in a regular and predictable manner will promote purposeful knowledge sharing, problem solving, and debate—grounded in the realities of women's lived experiences—and will facilitate increased application of MNH evidence, data, and metrics, fueling coordinated action, financing, tracking of progress, and mutual accountability, ultimately contributing to increased effective coverage of MNH care.

Tactics: Guided by a global steering committee with majority representation of thought leaders based in low- and middle-income countries:

- Convene stakeholders through a regular and predictable conference series focused on country priorities and needs
- Establish a multidirectional, dynamic knowledge hub driven by country priorities and needs
- Engage selected countries to ensure the knowledge hub and conference series are shaped by country priorities and needs and contribute to accelerating progress toward improved maternal, perinatal, and newborn health and well-being

AlignMNH provides an opportunity for multidirectional, dynamic sharing, but its successes will be defined by countries' access to actionable learning and new evidence, the development of equitable platforms and partnerships, and a focus on priorities relevant to countries. We recognize that we have much to learn to create effective spaces for solution-focused, action-oriented discussion and engaging experts and influencers outside of established, mainstream MNH communities. Sustained progress will only be possible with deliberate consideration and attention to underlying and social determinants of health and navigating complex adaptive systems, which must contribute to realizing the right of everyone to enjoy the highest attainable standard of physical and mental health. Maintaining a sense of urgency and momentum and continuing to be vigilant amidst these considerations will be a challenge, but without collective action, we risk not achieving the SDGs. Whatever strategies are pursued, they will only be possible with the active engagement of multiple stakeholders and concomitant commitments to universal health care access along the way.

We invite you to join us in this movement as we prepare to host a virtual Opening Forum of the AlignMNH Collective on April 20–21, 2021, and a biannual International Maternal and Newborn Health Conference in subsequent years with focused convening and discussions along the way. These events, along with the establishment of a related knowledge hub, will help set the stage for a decade of continued learning and collective action to drive progress for maternal, perinatal, and newborn health and well-being. Visit www.alignmnh.org to learn more.

## Notes

**Peer Reviewed**

**First published online:** April 19, 2021.** Cite this article as:** Gibson A, Noguchi L, Kinney MV, et al. Galvanizing collective action to accelerate reductions in maternal and newborn mortality and prevention of stillbirths. Glob Health Sci Pract. 2021;9(2):268-273. https://doi.org/10.9745/GHSP-D-20-00575
